# Mucous Membrane Pemphigoid, Bullous Pemphigoid, and Anti-programmed Death-1/ Programmed Death-Ligand 1: A Case Report of an Elderly Woman With Mucous Membrane Pemphigoid Developing After Pembrolizumab Therapy for Metastatic Melanoma and Review of the Literature

**DOI:** 10.3389/fmed.2018.00268

**Published:** 2018-09-27

**Authors:** Coralie Zumelzu, Marina Alexandre, Christelle Le Roux, Patricia Weber, Alexis Guyot, Annie Levy, Françoise Aucouturier, Sabine Mignot-Grootenboer, Frédéric Caux, Eve Maubec, Catherine Prost-Squarcioni

**Affiliations:** ^1^Department of Dermatology and Referral Center for Auto-Immune Bullous Diseases MALIBUL, Avicenne Hospital, AP-HP, University Paris 13, Bobigny, France; ^2^Department of Pathology, Avicenne Hospital, AP-HP, University Paris 13, Bobigny, France; ^3^Department of Immunology and Referral Center for Auto-Immune Bullous Diseases MALIBUL, Saint-Louis Hospital, AP-HP, Paris, France; ^4^Department of Immunology and Referral Center for Auto-Immune Bullous Diseases MALIBUL, Bichat Hospital, AP-HP, Paris, France; ^5^Department of Histology, UFR Léonard de Vinci, University Paris 13, Bobigny, France

**Keywords:** mucous membrane pemphigoid, bullous pemphigoid, melanoma, anti-programmed-death-1/death-ligand-1, immune checkpoints inhibitors, pembrolizumab, drug accountability study, adverse drug reaction

## Abstract

An 83-year-old patient developed erosions and a blister of the gingival mucous membrane, 6 months after discontinuation of the anti-programmed death-1 (anti PD-1) pembrolizumab therapy administered for 10 months for a metastatic melanoma. A diagnosis of mild mucous membrane pemphigoid (MMP) was made. Complete remission of MMP was rapidly obtained with minimal therapy (doxycycline). MMP remained in complete remission after a 3-month follow-up since discontinuation of the doxycycline therapy and no evidence of relapse of the melanoma was observed after a 14-month follow-up since discontinuation of the pembrolizumab therapy. The widespread use of anti PD-1 and anti-programmed death-ligand-1 (PD-L1) in several malignancies reveals new adverse events. MMP describes a group of chronic, inflammatory, mucous membrane-predominant, subepithelial auto-immune blistering diseases. It is clinically distinct from bullous pemphigoid another autoimmune blistering disease but shares some immunological similarities with it. Twenty-nine cases of bullous pemphigoid associated with anti PD-1/PD-L1 have been reported in the literature and one of MMP. Here, we described the case of a MMP developed after pembrolizumab and discussed the accountability of anti PD-1/PD-L1 in our case and the previous reported bullous pemphigoid and MMP cases using the Begaud system scoring.

## Background

Immune checkpoint inhibitors against programmed death-1 (anti PD-1) and programmed death-ligand 1 (anti-PD-L1) agents have revolutionized the treatment of metastatic melanoma and have shown encouraging promise in advanced solid tumors and hematological malignancies. However, these agents are associated with immune-related adverse events (IrAEs) that affect mainly the skin, hormone glands, liver and gastrointestinal tracts.

Indeed, up to 20% of treated patients may develop dermatological IrAEs. They are predominantly non-specific rashes and pruritus (1). Toxicities on buccal mucous membrane (MM) have also been described, including xerostomia, lichenoid reactions, and dysgeusia (2). Since 2015, an association between a treatment with anti PD-1/PD-L1 and bullous pemphigoid (BP) has been reported in 29 cases. One mucous membrane pemphigoid (MMP) case, an autoimmune bullous disease (AIBD) similar to BP, has also been described after pembrolizumab therapy.

Here, we report a second case of MMP that occurred 16 months after initiation of pembrolizumab therapy for a metastatic melanoma, discuss the association between MMP and melanoma, and review the literature on BP and MMP associated with anti PD-1/PD-L1.

## Case presentation

In 2014, an 83-year-old woman with no history of known autoimmune disease was diagnosed as having a right leg superficial spreading melanoma, initially T2b N0 M0. Eight months later, she developed iterative local and in transit cutaneous metastases on the same leg and she underwent four times surgical excision.

In 2016, at the fourth recurrence, surgery was not chosen. Baseline full-body computed tomography revealed no other metastasis (T2 N0 M1a). Mutation tested on a tumor sample excluded the presence of any BRAF mutation. Administration of pembrolizumab therapy was started at 2 mg/kg every 3 weeks, resulting in complete remission (CR) within 3 months (cycle 4). In March 2017, after 14 cycles, she remained in CR, and the pembrolizumab therapy was stopped at her request.

**Figure 1:**
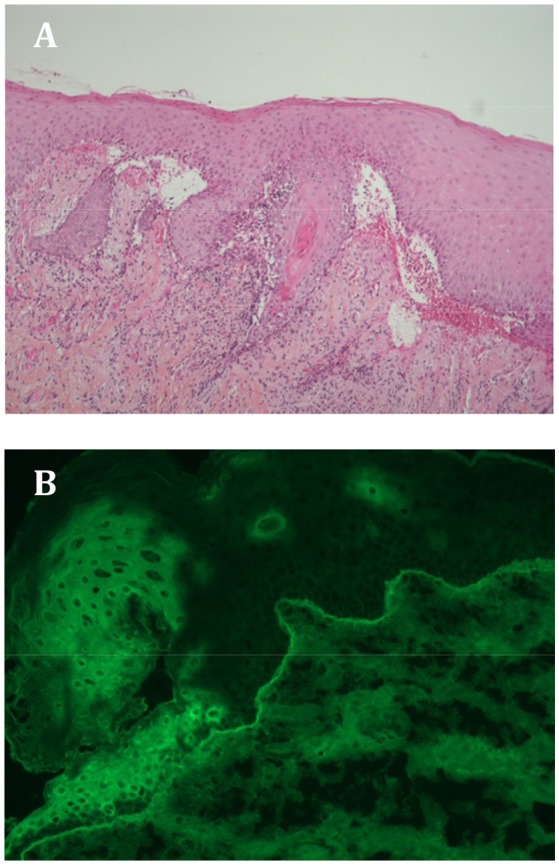
**(A)** Histological examination of a gingival biopsy showing subepithelial cleavage with overlying intact epithelium. A moderate perivascular infiltration can be observed consisting of lymphocytes and histiocytes, and no lichenoid infiltrate. **(B)** Direct immunofluorescence microscopic image showing linear IgG (++) and C3 (++) immune deposits along the basement membrane zone on the gingival biopsy.

**Figure 2:**
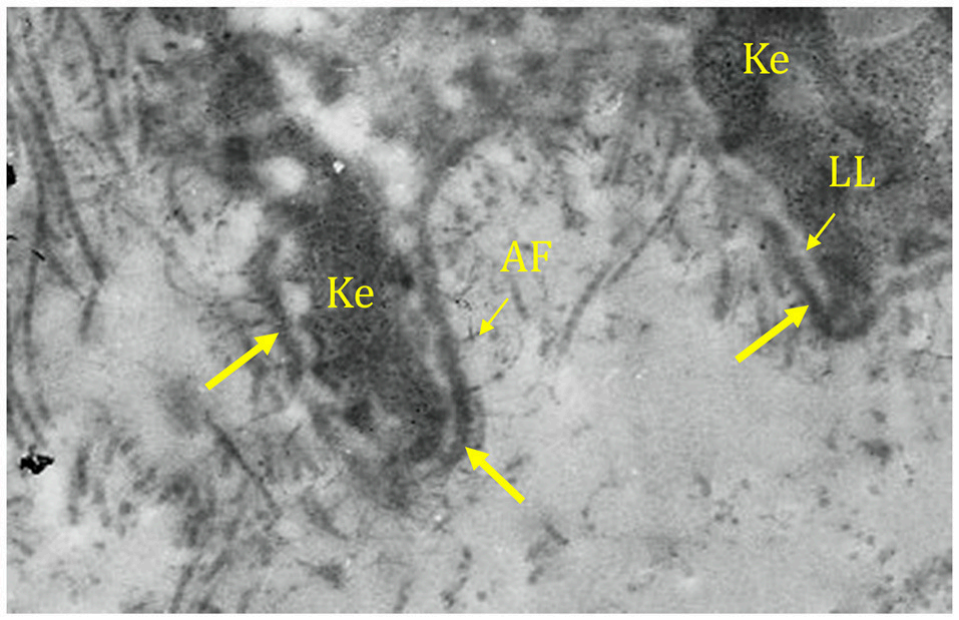
Direct immunoelectron microscopy showing immune deposits (arrow) strictly localized in the lamina densa. Ke, keratinocyte; LL, lamina lucida; AF, anchoring fibril.

In October 2017, 6 months after pembrolizumab discontinuation, she complained of oral pain and was referred to our hospital. Clinical examination revealed gingivitis with one tense blister, a large pseudomembrane-covered erosion with a tweezers sign, an atrophy and pseudo lichenoid lesions. Other MM and skin were not involved. Gingival biopsy showed a subepithelial cleavage with the overlying intact epithelium (Figure 1A). A moderate perivascular infiltration consisting of lymphocytes and histiocytes was observed, with no lichenoid infiltrates. Direct immunofluorescence (DIF) microscopy revealed linear IgG (++) and C3 (++) immune deposits along the basement membrane zone (BMZ) (Figure 1B). Standard indirect immunofluorescence (IIF) microscopy on rat esophagus failed to detect circulating anti-BMZ antibodies. A diagnosis of mild MMP was made. Further immunological investigations demonstrated that the immune deposits identified using direct immunoelectron microscopy (IEM) were strictly localized in the lamina densa (Figure 2), a site consistent with autoantibodies against the laminin 332 or the C-terminal extremity of BP180 antigen (BP180). IIF on salt-split skin, immunoblot using amniotic extracts, and enzyme-linked immunosorbent assays (ELISAs) with BP180-NC16A epitope and BP230 antigen (BP230) had negative results.

Doxycycline therapy (100 mg/day) and mouth washes with corticosteroid (betamethasone 2 mg) three times daily were initiated, which led to the control of the MMP within 2 weeks and CR under this minimal therapy in 6 weeks. After 3 months of treatment with doxycycline therapy, the patient decided on her own to discontinue it, and no MMP relapse had occurred 3 months later. No clinical or radiological evidence of relapse of the melanoma was observed on computed tomography imaging after a 14-month follow-up since discontinuation of the pembrolizumab therapy.

## Discussion

Our case raises the question of an association between MMP and melanoma, or between MMP/BP and pembrolizumab administration.

MMP encompasses a group of AIBDs clinically defined by the predominance of MM lesions over skin lesions (3, 4), and healing of its lesions leads to characteristic cicatricial scarring. The buccal involvement is the most frequent, followed in order of decreasing frequency by ocular, nasal, nasopharyngeal, anogenital, skin, laryngeal, and oesophageal involvements. The ocular, laryngeal, and oesophageal involvements can cause severe impairment or even death.

Our patient had a typical MMP, except the age at MMP onset, in a mild form because of the purely buccal involvement. Thus she was considered as a “low-risk patient” with few tendencies of scarring and required minimal therapy with doxycycline and topical steroids (3). Her MMP was controlled in 2 weeks, in CR on minimal therapy in 6 weeks, and in CR off treatment in 3 months. She developed mild cicatricial lesions of her gingival MM and did not relapse during follow-up. Rapid clinical improvement after only a short course of treatment is unusual in MMP.

MMP results from the activity of autoantibodies directed against BMZ antigens. The main autoantibody target is BP180, with the sera of most MMP patients reacting with its C-terminal domain (BP180-C term), combined or not with reactivity against the BP180-NC16A epitope and BP230 (5, 6). Other target antigens associated with a clinical MMP phenotype have been characterized molecularly, including the following: laminin 332, both α6β4-integrin subunits, and type VII collagen (7), respectively defining laminin 332-MMP, α6β4-integrin MMP, and MM epidermolysis bullosa acquisita. Autoreactive T lymphocytes are thought to also play a key role in the pathogenesis of MMP, particularly in the fibrosing process (8–15).

Our patient had a linear deposition of IgG/C3 along the epithelial BMZ on DIF microscopy and on the lamina densa on direct IEM, a location consistent with targeting of the C-terminal extremity of BP180 or laminin 332 by autoantibodies (16, 17). No circulating antibodies against BMZ antigens were detected by serological studies, notably BP180-NC16A ELISA, as in 49% of MMP in a recent series (18).

An association between laminin 332-MMP and malignancy was first reported in 1998 but is currently controversial. On one hand, 21 cases of laminin 332-MMP have been reported in association with cancer, including 15 reviewed by Sadler in 2007 (19) and six case reports after 2007 (20–25). An increased risk of solid cancers as compared with the general population was reported by two authors (26, 27), higher in the first year following the laminin 332-MMP diagnosis. On the other hand, no significant correlation was found between laminin 332 reactivity and the proportion of patients with an associated internal cancer in three recent serological studies of MMP (28–30).

Anyway, no association between laminin 332-MMP and melanoma has been reported (31).

In our patient, who had a possible laminin 332-MMP, a link between MMP and melanoma seems unlikely, as the first incidence occurred 3 years after the second and the latter was in CR.

We examined the intrinsic accountability of pembrolizumab therapy on MMP induction in our patient with metastatic melanoma because of its extrinsic accountability based on the following reports: (i) MMP and BP have immunological similarities (7); (ii) intrinsic accountability of anti PD-1/PD-L1 treatments on BP induction: 27 BP have been reported as case reports or short series (32–49) and two BPs listed as adverse drug reaction in two large trials with anti PD-1 (50, 51); (iii) recently, one pembrolizumab-associated MMP case report (52), and (iv) some of the anti PD-1/PD-L1-associated BPs had atypical clinical phenotypes (33–35, 42, 47).

Although the clinical characteristics of MMP differ from those of BP typified by the absence of MM lesions, absence of predominant head-and-neck involvement, absence of scars, and older age at onset (>70 years) (53), MMP and BP share physiopathological features; BP result from the activity of autoantibodies directed against BP230 and BP180, such as most MMPs. However, the sera of most patients with BP react with the BP180-NC16A epitope, contrary to those of patients with MMP.

The melanoma treatment has been revolutionized by innovative immunomodulation drugs that break tolerance. The main treatment targets for melanoma are cytotoxic T-lymphocyte antigen-4 and PD-1. Ipilimumab, a monoclonal antibody against cytotoxic T-lymphocyte antigen-4, was the first drug to demonstrate a benefit in overall survival in a randomized controlled phase 3 study of patients with advanced melanoma (54) and to be approved by the Food and Drug Administration (FDA) and European Medicines Agency (EMA). The anti-PD-1s and anti-PD-L1s are remarkably more effective in terms of response and overall survival rates than ipilimumab. The response rate with anti-PD-1 reaches 40% in melanoma, and the 5-year overall survival rate for naive patients is close to 40% (55). Moreover, anti-PD-1s are less toxic in terms of IrAEs. In 2014, the FDA approved the anti-PD-1 antibodies pembrolizumab and nivolumab for advanced melanoma and, in 2015, the combination of ipilimumab and nivolumab.

PD-1 is a negative co-stimulatory receptor, which downregulates excessive immune responses by binding to its ligands, PD-L1 and PD-L2. This receptor is expressed mainly on activated T cells. In tumor tissue, binding of PD-1 inhibits effector T-cell function, which leads to exhausted T cells and suppression of the antitumor immune response (56). Pembrolizumab and nivolumab on one hand and atezolizumab and durvalumab on the other hand are selective humanized monoclonal antibodies that bind respectively to PD-1 and PD-L1 and thus block the interaction between PD-1 and its ligands, which leads to stimulatory effects on T cells.

B cells also express PD-1, and inhibition of PD-1 expression can directly activate B cells in a T-cell-independent manner (57). Lastly, it appears that anti PD-1 reduce regulatory T-cell activity (58), resulting in decreased tolerance and development of autoimmunity. All these mechanisms can be involved in the induction of MMP as soon as B and T cells played a central role in the MMP pathogenesis.

The potential drug induction of AIBD has been known for decades (18, 59–66). Recently, between 2015 and 2018, 18 case reports or small series described 27 patients who developed BP while receiving anti PD-1/PD-L1 therapy, including three with negative DIF (35, 37) or without DIF confirmation (47) but with typical clinical presentations (Tables 1, 2). Fourteen patients received anti PD-1 nivolumab therapy (patients 2, 4, 7, 9, 10, 13, 15, 18, 20, 22, 23, and 25–27) (33, 35, 38, 42, 44, 46, 47, 49), 11 received anti PD-1 pembrolizumab therapy (patients 1, 5, 6, 8, 11, 12, 14,16, 19, 21, and 24) (32, 34–37, 39, 40, 43, 45, 48), one received anti-PD-L1 durvalumab therapy (patient 3) (33), and one received anti-PD-L1 atezolizumab therapy (patient 17) (41). Six of the 27 patients received ipilimumab therapy before anti PD-1, one received an association of ipilimumab and nivolumab therapy, and one received ipilimumab therapy after treatment with nivolumab. Two additional BP cases within a large series were simply mentioned, without any clinical description (50, 51). Recently, one MMP case treated with pembrolizumab therapy for a metastatic Merkel cell carcinoma has also been reported (53). Notably, the outcomes of the patients with melanoma were better [only 5 (33%) out of 15 cases in which the information were available, had a progressive disease] than reported in general anti-PD-1 treatment of melanoma (55). Other studies reported that objective response rate was significantly higher in patients with melanoma who experienced nivolumab-related adverse events (67).

**Table 1:** Case reports of anti-PD1/PD-L1-treated bullous pemphigoid patients.

**Patients**	**First author, year (ref N°)**	**Sex/age (year)**	**Cancer type**	**Ipilimumab before anti-PD1**	**Other therapies before anti-PD1**	**Anti-PD1/-PD-L1**	**Dose^a^**	**1st anti PD1/PD-L1 dose to BP onset (weeks)**
**CASE REPORTS**								
1	Carlos et al. (32)	M/75	Melanoma	Yes	Chemotherapy	Pembrolizumab	10 mg/kg	22
2	Naidoo et al. (33)	M/80	Melanoma	Yes	No	Nivolumab	NR	24
3		F/78	Melanoma	Yes	No	Durvalumab	NR	52
4		M/85	Lung SCC	No	Chemotherapy	Nivolumab	NR	18
5	Hwang et al. (34)	M/68	Melanoma	No	No	Pembrolizumab	10 mg/kg	78
6		M/72	Melanoma	No	No	Pembrolizumab	10 mg/kg	27
7	Jour et al. (35)	M/63	Tongue SCC	No	Radiation, chemotherapy, erlotinib	Nivolumab	3 mg/kg	8
8		M/68	Melanoma	No	No	Pembrolizumab^b^	2 mg/kg	16.4
9		F/74	Urothelial cancer	Yes + nivolumab	No	Nivolumab	3 mg/kg	16
10		F/73	Adenocarcinoma	No	Radiation, chemotherapy	Nivolumab	3 mg/kg	6
11	Mochel et al. (36)	M/63	Melanoma	No	No	Pembrolizumab	NR	84
12	Lomax et al. (37)	F/82	Melanoma	Yes	No	Pembrolizumab	NR	32
13	Damsky et al. (38)	F/77	Lung adenocarcinoma	No	No	Nivolumab	3 mg/kg	6
14	Bandino et al. (39)	M/73	Melanoma	No	No	Pembrolizumab	2 mg/kg	18
15		M/90	Melanoma	No	No	Pembrolizumab, nivolumab^c^	2 mg/kg, 3 mg/kg	24
16	Rofe et al. (40)	F/56	Melanoma	Yes	No	Pembrolizumab	2 mg/kg	24
17	Russo et al. (41)	M/58	Lung adenocarcinoma	No	Chemotherapy, bevacizumab	Atezolizumab	1200 mg	60
18	Sowerby et al. (42)	M/80	Lung adenocarcinoma	No	No	Nivolumab	3 mg/kg	80
19	Parakh et al. (43)	M/42	Melanoma	Yes	Radiation, chemotherapy, dabrafenib, trametinib	Pembrolizumab	2 mg/kg	44
20	Kwon et al. (44)	M/60	Renal cell carcinoma	No	Chemotherapy	Nivolumab	3 mg/kg	12
21	Wada et al. (45)	M/65	Melanoma	No	No	Pembrolizumab	2 mg/kg	51
22	Kuwatsuka et al. (46)	M/35	Melanoma	No^d^	No	Nivolumab^d^	NR	50
23	Anastasapoulou et al. (47)	M/48	Melanoma	No	No	Nivolumab	3 mg/kg	31
24	Amber et al. (48)	F/82	Melanoma	No	No	Pembrolizumab	2 mg/kg	27
25	Le Naour et al. (49)	M/66	Choroidal melanoma	No	No	Nivolumab	NR	28
26		M/78	Melanoma	No	No	Nivolumab	NR	16
27		F/68	Non-small-cell lung cancer	No	No	Nivolumab	NR	16
**LARGE SERIES**								
28	Muro et al. (50)	NR	Gastric cancer	NR	NR	Pembrolizumab	10 mg/kg	NR
29	El Khoueiry et al. (51)	NR	Hepatocellular carcinoma	NR	NR	Nivolumab	NR^e^	NR

a *Pembrolizumab, atezolizumab were administered every 3 weeks and nivolumab, durvalumab every 2 weeks*.

b *+ dabrafenib (150 mg) and trametinib (2 mg) after 3 cycles*.

c *Pembrolizumab 4 cycles switch to nivolumab + radiation*.

d *Bullous pemphigoid occured during ipilimumab, administered after nivolumab*.

e *Nivolumab 0.1-10 mg/kg every 2 weeks in the dose-escalation phase, nivolumab 3 mg/kg every 2 weeks in the dose-expansion phase*.

*PD1, Programmed-Death-1; PD-L1, Programmed-Death-Ligand-1; SCC, squamous Cell Carcinoma; NR, Not reported*.

**Table 2:** Clinical, immunological, and evolutive data in 27 of the 29 anti-PD1/PD-L1-treated BP patients reported in the literature.

	**Clinical data**			**Immunological data**		**Treatment**			**Dechallenge**					**Outcome at last visit**	
	**(Extensive/moderate/localized)**	**Atypical lesions (No/Yes, atypical site)**	**Neurological disorder (No/Yes)**	**DIF**	**ELISA BP180/BP230**	**Systemic CS**	**Local CS**	**Others**	**Yes/No**	**Time between BP onset and dechallenge (weeks)**	**Because of BP/cancer**		**Re- challenge Yes/No**	**BP**	**Cancer**
1	Extensive	No	NR	+	NR	Yes	Yes	No	Yes	−4.3	No/	Yes, PD	No	Improvement	PD death
2	NR	Yes (buccal MM)	NR	+	+/–	Yes	Yes	Antihistamines, tacrolimus, oral ointment	Yes	28	Yes/	No	No	Improvement but peaked at each dose	CR
3	Moderate	Yes (buccal MM)	NR	+	+/+	No	Yes	No	Yes	0	Yes/	No	No	Improvement	prolonged PR
4	Extensive	No	NR	+	–/–	Yes	Yes	No	Yes	0	Yes/	No	No	Stable	prolonged SD
5	Extensive	Yes (buccal MM and face)	NR	+	NR	No	Yes	No	Yes	3	No/	Yes, prolonged PR	No	Initial response then relapse then NR	prolonged PR
6	Extensive	Yes (buccal MM and scalp)	NR	+	NR	Yes	Yes	Cyclins, methotrexate	Yes	>21	Yes/	Yes, PD	No	Initial response then relapse	PD death
7	Moderate	Yes (buccal MM, neck and face)	NR	+	NR	Yes	Yes	No	Yes	0	Yes/	No	Yes	Relapse at rechallenge—resolution at dechallenge	PD
8	Extensive	No	NR	+	NR	Yes	Yes	No	Yes	0	Yes/	No	No	Improvement	PD death
9	Extensive	No	NR	+	NR	Yes	Yes	No	Yes	0	Yes/	No	No	Remission	PR
10	Moderate	No	NR	–	NR	Yes	Yes	Cyclins, niacinamide	Yes	7	Yes/	Yes, PD	No	Improvement then relapse	PD
11	Moderate	No	No	+	+/–	Yes	Yes	No	Yes	12	No/	Yes, near CR	No	Improvement	CR
12	Extensive	No	NR	–	NR	Yes	Yes	Loratadine, prometazine	Yes	0	Yes/	No	No	Improvement but pruritus	CR
13	Extensive	No	depression	+	+/–	Yes	Yes	Omalizumab	Yes	0	Yes/	No	Yes	Controlled	NR
14	Moderate	No	NR	+	NR	No	No	Cyclins, niacinamide	Yes	6	No/	No	No	Slowly Improvement	NR
15	Localized	No	NR	+	NR	Yes	Yes	No	Yes	12	No/	Yes, CR	No	Improvement	CR
16	Extensive	No	NR	+	+/–	Yes (bolus)	Yes	Methotrexate	Yes	0	Yes/	Yes, PD	No	Improvement then relapse	CR
17	Extensive	No	NR	+	+/–	Yes	No	Cyclins	Yes	0	Yes/	No	No	Remission	CR
18	Localized	Yes (buccal MM)	NR	+	+/–	Yes	No	No	Yes	0	Yes/	No	No	Remission after rituximab	CR
19	Extensive	No	Paraplegia	+	NR	Yes	Yes	Cyclins, nicotinamide	Yes	0	Yes/	No	No	Remission	PR
20	Extensive	No	NR	+	NR	Yes	Yes	No	Yes	0	Yes	No	No	Remission	NR
21	Moderate	No	NR	+	+/–	Yes	Yes	No	NR	NR	Nd/	Nd	No	Controlled	CR
22	Moderate	No	NR	+	+/–	No	Yes	No	Yes	< –6	No/	Yes,PD	No	Remission	NR
23	Extensive	Yes, (face and neck)	NR	Nd	NR	Yes	No	No	Yes	−12	No/	Yes, PD	No	Remission	PD
24	Localized (lower legs) then extensive	No	NR	+	+	Yes	Yes	No	No	No	Nd	Nd	No	Controlled	NR
25	Localized	No	NR	+	+	Yes	No	No	No	No	Nd	Nd	No	Controlled	PR
26	Localized	No	NR	+	NR	Yes	No	No	No	4	No	Yes, PD	No	Controlled	PD/palliative care
27	Extensive	No	NR	+	NR	Yes	Yes	No	No	0	No	PD	No	Remission	PD

*PD1, Programmed Death-1; PD-L1, Programmed Death Ligand-1; BP, bullous pemphigoid; DIF, direct immunofluorescence; ELISA, enzyme-linked immunosorbent assay; BP180, BP180 antigen; BP230, BP 230 antigen; CS, corticosteroid; +, positive; –, negative; NR, not reported; PD, progression disease; MM, mucous membrane; CR, complete remission; PR, partial response; SD, stable disease; ND, not determinated*.

The overall characteristics of these patients with anti PD-1/PD-L1-associated BPs were as follows: eight women and 19 men (female-to-male sex ratio, 0.4), median age of 68 years (35–90 years) at the time of BP diagnosis, and a median interval of 24 (6–84) weeks between starting anti PD-1/PD-L1 therapy (challenge) and BP onset. This time was significantly shorter (median, 16 weeks; range, 6–80 weeks) with nivolumab than with pembrolizumab (median, 27 weeks; range, 16–84 weeks) (Wilcoxon rank sum test with continuity correction: *p* = 0.023).

Pruritus was a prominent feature of most cases (68). Administration of anti PD-1/PD-L1 agents was discontinued (dechallenge) in 21 of the 27 patients (patients 1–14, 16–20, 22, 23, and 27) because of the evolution of BP and/or cancer and continued in five patients (no dechallenge, patients 1, 15, and 24–26). This information is unknown in one patient (patient 21). BP treatment with systemic steroids was required in all but four patients (patients 3, 5, 14, and 22). AntiPD-1/PD-L1 was reintroduced in two patients (rechallenge; patients 7 and 13). The reasons of the dechallenge, and the outcome after dechallenge or rechallenge are detailed in Table 2.

The comparison with the “usual” BPs (7, 69) highlights particularities in these anti PD-1/PD-L1-associated BPs as follows: a predominance of males (female-to-male sex ratio, 0.4 vs. 1.5), younger age [mean, 69 years; median, 68 years (range, 35–90 years) vs. mean, 83 years], no evidence of neurological disorders, more extensive diseases (52 vs. 41%), and no circulating autoantibodies against BP230 except in one patient (8 vs. 60–70%), while 91% of the tested sera had circulating autoantibodies against BP180-NC16A (vs. 80–90%). Moreover, seven cases had atypical clinical phenotypes, as head and neck involvement or mucosal lesions (33–35, 42, 47), raising doubts about the diagnosis of BP (53).

The patient with pembrolizumab-associated MMP reported by Haug et al. was a 62-year-old man who developed a pure buccal MMP after 13 weeks of pembrolizumab therapy. He had circulating autoantibodies targeting the C-terminal extremity of BP180. Pembrolizumab was discontinued at the time of MMP onset, and he was successfully treated with doxycycline and topical steroid, as in our case.

In our patient, MMP developed after 14 cycles and 24 weeks of pembrolizumab discontinuation, that is, 66 weeks after starting the treatment. Overall, IrAE induced by anti PD-1/PD-L1 agents usually appeared between 1 week and several months after starting immunotherapy (2). Among the 27 cases of anti PD-1/PD-L1-associated BPs, four had a long delay (>60 weeks) between starting anti PD-1/PD-L1 therapy and onset of BP (patients 5, 11, 17, and 18) (34, 36, 41, 42). Similarly to our patient, three patients developed a BP 4, 12, and 12 weeks after discontinuation of the anti PD-1 therapy (patients 1, 22, and 23, respectively) (32, 46, 47). This could be explained by the durable activity of anti PD-1/PD-L1 on immunity (70, 71).

Lastly, we assessed the intrinsic accountability score of anti PD-1/PD-L1 in AIBD induction for our patient with MMP, and *a posteriori* for the 27 BPs and MMP cases that have been reported using the Begaud scoring system (terms in bold type), updated in 2011 (72). The present **challenge** was the treatment of a malignancy by using an anti PD-1/PD-L1. Patients with a malignancy who started treatment with anti PD-1/PD-L1 before MMP/BP onset may have a **suggestive** or **compatible** challenge. The **dechallenge** was the discontinuation of the treatment. Outcome after **dechallenge** or **no dechallenge** may be **suggestive** (if BP is controlled with **dechallenge** or worsened **without dechallenge**), conversely **non-suggestive** (if BP worsened after **dechallenge** or controlled unless **without dechallenge**), and **inconclusive** (without details on BP evolution or continued treatment).

The **rechallenge** was the reintroduction of the treatment. It may be **positive** (R+) or **negative** (R–) or **not done** (R0). The **chronological** scoring (combining status of **challenge**, **dechallenge**, and **rechallenge**) may be C1, **doubtful**; C2, **plausible**; and C3, **likely**. The **symptomatological** scoring may be S1, **doubtful**; S2, **plausible**; and S3, **likely**. Lastly, the **intrinsic accountability** scoring [combining **chronological** (C) and **symptomatological** (S) scores] may be I1 (C1S1), I2 (C1S2 or C2S1), I3 (C2S2), I4 (C1S3 or C3S1), I5 (C2S3 or C3S2), or I6 (C3S3) (see detailed results in Supplementary Data, Table S1).

The Begaud system scoring indicates that the possibility of anti PD-1/PD-L1 as a BP triggering factor is mostly low: only 10 (four with pembrolizumab, five with nivolumab, and one with durvalumab) of the 27 patients with BP were given high accountability scores [I5 for three patients (patients 2, 6, and 7), I4 for five (patients 8, 9, 14, 20, and 27), I3 for two (patients 3 and 5)], while 17 (seven with pembrolizumab, nine with nivolumab, and one with atezolizumab) had low accountability [I2 for six (patients 10, 12, 17, 18, 19, and 23) and I1 for 11 (patients 1, 4, 11, 13, 15, 16, 21, 22, and 24–26)]. One patient with BP had a positive rechallenge, and another had a negative rechallenge.

The Begaud system scoring indicates that the intrinsic accountability score was I4 for the MMP case that was reported but I1 for our patient with MMP.

In all the MMP/BP patients, the low score was essentially due to the long time between anti PD-1 introduction and AIBD onset (compatible challenge) and/or a non-suggestive dechallenge. Indeed, it could be the consequence of long delay of action of anti PD-1/PD-L1. A non-suggestive dechallenge may not be an argument against the accountability of a long-acting drug, and at the end, the Begaud scoring system may not be suitable for assessing the accountability of drugs with prolonged therapeutic effect.

In our patient, the eventuality of a rechallenge did not occur because the melanoma and MMP remained in CR. As an anti PD-1-induced MMP is possible, a rechallenge would theoretically expose her to a risk of MMP relapse in a potentially more serious form. Indeed, MMP can involve ocular, nasopharyngeal, laryngeal, esophageal, genital, or anal MM, sites that have a high likelihood of scarring, which is associated with loss of function. On the other hand and contrary to literatures on adverse drug reactions, a negative rechallenge with anti PD-1 has already been reported (38, 73).

In conclusion, we report the case of a patient who developed a mild MMP, possibly induced by anti PD-1 rather than by melanoma. We cannot also exclude that the MMP could be triggered by aging, malignancy, and pembrolizumab acting in concert or developed quite independently. MMP was rapidly controlled by a minimal treatment, raising the question of reintroduction of anti PD-1 if the melanoma relapses. With the increasing use of immunotherapies for various malignancies, clinicians should be alert for this new anti PD-1-induced IrAE, which is related to BP but potentially more severe. Long-term clinical follow-up is warranted owing to delayed adverse events, even after discontinuation of anti PD-1 inhibitors. Lastly the Begaud system scoring applied to our patient and previous reported cases with anti-PD-1/PD-L1 related BP/MMP indicates a low intrinsic accountability score in most of the patients suggesting it may not be suitable for assessing the accountability of drugs with prolonged therapeutic effect. Development of another specific assessment might be necessary.

## Ethics statement

A written consent has been obtained from the patient.

## Author contributions

CZ and CP-S conceived and designed the study. CZ, MA, CL, PW, AG, and EM collected clinical data. AL and CP-S conducted the histological studies and FA and SM-G the immunological ones. CZ wrote the first draft of the manuscript. CP-S rewrote sections of the manuscript. EM and FC corrected the final version. All authors contributed to manuscript revision, and read and approved the submitted version.

### Conflict of interest statement

The authors declare that the research was conducted in the absence of any commercial or financial relationships that could be construed as a potential conflict of interest.

## Abbreviations

AIBD: autoimmune bullous disease
anti PD-1: anti-Programmed-Death-1
anti-PD-L1: anti-Programmed-Death-ligand-1
BMZ: basement membrane zone
BP: bullous pemphigoid
BP180: BP180 antigen
BP230: BP230 antigen
CR: complete remission
DIF: direct immunofluorescence
ELISA: enzyme-linked immunosorbent assay
FDA: Food and Drug Administration
IEM: immunoelectron microscopy
IIF: indirect immunofluorescence
IrAEs: immune-related adverse events
MM: mucous membrane(s)
MMP: mucous membrane pemphigoid.
