# Supplementary material for: Mucous Membrane Pemphigoid, Bullous Pemphigoid, and Anti-programmed Death-1/ Programmed Death-Ligand 1: A Case Report of an Elderly Woman With Mucous Membrane Pemphigoid Developing After Pembrolizumab Therapy for Metastatic Melanoma and Review of the Literature
Source: Front Med (Lausanne). 2018 Sep 27;5:268. doi: 10.3389/fmed.2018.00268 (PMC6170650; doi:10.3389/fmed.2018.00268)
Supplement: Supplementary file 1 [file Data_Sheet_1.docx]

| **Table S1.** Anti-PD1/ PD-L1 accountability scores for BP induction in the 27 BP patients of the literature. | | | | | |  |  |  |  |  |  |  |
| --- | --- | --- | --- | --- | --- | --- | --- | --- | --- | --- | --- | --- |
|  |  |  |  |  |  |  |  |  |  |  |  |  |
|  |  |  |  |  |  |  | **Dechallenge** | |  | **Begaud's accountability scores^b^** | | |
| **Patients** | **First author,  year (ref N°)** | **Sex/age (year)** | **1^st^ anti PD1/  PD-L1 dose  to BP onset** | **Discontinuation  anti PD1/PD-L1  before BP onset No/Yes, time** | **Challenge^a^** | **Atypical lesions (No/Yes,  atypical site)** | **Yes/No** | **Outcome** | **Rechallenge (R0/R^+^/R^–)^** | **Chronological  score  (C1-C3)** | **Semiological  score  (S1-S3)** | **Intrinsic accountability  I0-I6** |
|  |  |  |  |  |  |  |  |  |  |  |  |  |
| **Nivolumab** |  |  |  |  |  |  |  |  |  |  |  |  |
| 20 | Kwon et al, 2017 (43) | M/60 | 12 We | No | Suggestive | No | Yes | Suggestive | R0 | C3 | S1 | I4 |
| 9 | Jour et al, 2016 (34) | F/74 | 16 We | No | Suggestive | No | Yes | Suggestive | R0 | C3 | S1 | I4 |
| 27 | Le Naour et al, 2018 (48) | F/68 | 16 We | No | Suggestive | No | Yes | Suggestive | R0 | C3 | S1 | I4 |
| 2 | Naidoo et al, 2016 (32) | M/80 | 24 We | No | Suggestive | Yes  (buccal MM) | Yes | Suggestive | R0 | C3 | S2 | I5 |
| 10 | Jour et al, 2016 (34) | F/73 | 6 We | No | Suggestive | No | Yes | Inconclusive | R0 | C2 | S1 | I2 |
| 4 | Naidoo et al, 2016 (32) | M/85 | 18 We | No | Suggestive | No | Yes | Not suggestive | R0 | C1 | S1 | I1 |
| 23 | Anastasapoulou et al, 2018 (46) | M/48 | 31 We | Yes, 19 We | Suggestive | Yes,  (face and neck) | Yes | Not suggestive | R0 | C1 | S2 | I2 |
| 22 | Kuwatsuka et al, 2018 (45) | M/35 | 50 We | Yes, 36 We | Compatible | No | Yes | Not suggestive | R0 | C1 | S1 | I1 |
| 18 | Sowerby et al, 2017 (41) | M/80 | 80 We | No | Compatible | Yes (buccal MM) | Yes | Not suggestive | R0 | C1 | S2 | I2 |
| 15 | Bandino et al, 2017 (38) | M/90 | 12 We | No | Suggestive | No | No | Not suggestive | R0 | C1 | S1 | I1 |
| 26 | Le Naour et al, 2018 (48) | M/78 | 16 We | No | Suggestive | No | No | Not suggestive | R0 | C1 | S1 | I1 |
| 25 | Le Naour et al, 2018 (48) | M/66 | 28 We | No | Suggestive | No | No | Not suggestive | R0 | C1 | S1 | I1 |
| 7 | Jour et al, 2016 (34) | M/63 | 8 We | No | Suggestive | Yes (buccal MM,  neck and face) | Yes | Suggestive | R+ | C3 | S2 | I5 |
| 13 | Damsky et al, 2016 (37) | F/77 | 6 We | No | Suggestive | No | Yes | Suggestive | R- | C1 | S1 | I1 |
|  |  |  |  |  |  |  |  |  |  |  |  |  |
| **Pembrolizumab** | |  |  |  |  |  |  |  |  |  |  |  |
| 8 | Jour et al, 2016 (34) | M/68 | 16 We | No | Suggestive | No | Yes | Suggestive | R0 | C3 | S1 | I4 |
| 14 | Bandino et al, 2017 (38) | M/73 | 18 We | No | Suggestive | No | Yes | Suggestive | R0 | C3 | S1 | I4 |
| 6 | Hwang et al, 2016 (33) | M/72 | 27 We | No | Suggestive | Yes (buccal MM  and scalp) | Yes | Suggestive or Inconclusive | R0 | C3 | S2 | I5 |
| 12 | lomax et al, 2016 (36) | F/82 | 32 We | No | Suggestive | No | Yes | Inconclusive | R0 | C2 | S1 | I2 |
| 16 | Rofe et al, 2017 (39) | F/56 | 24 We | No | Suggestive | No | Yes | Not suggestive | R0 | C1 | S1 | I1 |
| 19 | Parakh et al, 2017 (42) | M/42 | 44 We | No | Compatible | No | Yes | Suggestive | R0 | C2 | S1 | I2 |
| 5 | Hwang et al, 2016 (33) | M/68 | 65 We | No | Compatible | Yes (buccal MM  and face) | Yes | Suggestive | R0 | C2 | S2 | I3 |
| 11 | Mochel et al, 2016 (35) | M/63 | 84 We | No | Compatible | No | Yes | Not suggestive | R0 | C1 | S1 | I1 |
| 1 | Carlos et al, 2015 (31) | M/75 | 22 We | Yes, 4 We | Suggestive | No | No | Not suggestive | R0 | C1 | S1 | I1 |
| 24 | Amber et al, 2018 (47) | F/82 | 27 We | No | Suggestive | No | No | Not suggestive | R0 | C1 | S1 | I1 |
| 21 | Wada et al, 2017 (44) | M/65 | 51 We | No | Compatible | No | NR | Inconclusive | R0 | C1 | S1 | I1 |
|  |  |  |  |  |  |  |  |  |  |  |  |  |
| **Others** |  |  |  |  |  |  |  |  |  |  |  |  |
| 3^c^ | Naidoo et al, 2016 (32) | F/78 | 52 We | No | Compatible | Yes (buccal MM) | Yes | Suggestive | R0 | C2 | S2 | I3 |
| 17^d^ | Russo et al, 2017 (40) | M/58 | 60 We | No | Compatible | No | Yes | Suggestive | R0 | C2 | S1 | I2 |
|  |  |  |  |  |  |  |  |  |  |  |  |  |
|  |  |  |  |  |  |  |  |  |  |  |  |  |
| a Suggestive, time to onset ≤32 weeks; Compatible, time to onset > 44 weeks. | | | | |  |  |  |  |  |  |  |  |
| b Chronological score: C1, doubtful; C2, plausible; C3, likely. Symptomatological scoring: S1, doubtful; S2, plausible; S3, likely. | | | | | | |  |  |  |  |  |  |
| Intrinsic accountability scoring (combining chronological (C) and symptomatological (S) scores):I1 (C1S1), I2 (C1S2 or C2S1), I3 (C2S2), I4 (C1S3 or C3S1), I5 (C2S3 or C3S2), I6 (C3S3) (71). | | | | | | | | | | | | |
| ^c^ under durvalumab | |  |  |  |  |  |  |  |  |  |  |  |
| ^d^ under atezolizumab | |  |  |  |  |  |  |  |  |  |  |  |
| Abbreviations: wk, week; R0, no rechallenge; R+, positive rechallenge; R–, negative rechallenge; NR, not reported. | | | | | |  |  |  |  |  |  |  |
