# Supplementary material for: Mucous Membrane Pemphigoid, Bullous Pemphigoid, and Anti-programmed Death-1/ Programmed Death-Ligand 1: A Case Report of an Elderly Woman With Mucous Membrane Pemphigoid Developing After Pembrolizumab Therapy for Metastatic Melanoma and Review of the Literature
Source: Front Med (Lausanne). 2018 Sep 27;5:268. doi: 10.3389/fmed.2018.00268 (PMC6170650; doi:10.3389/fmed.2018.00268)
Supplement: Supplementary file 2 [file Data_Sheet_2.docx]

**Supplementary data**

Accountability criteria were analyzed and scored using Begaud’s system, updated in 2011 (terms in **bold type**, Miremont-Salamé G). Detailed results are reported in Table S1 of Supplementary Material.

**Extrinsic accountability** was rated B2 (sparse and/or unreliable publications) for our patient and all the anti PD-1/PD-L1-treated patients with BP in the literature.

The present **challenge** was the treatment of a malignancy by using an anti PD-1/PD-L1; 14 patients with BP were treated with nivolumab (anti PD-1), 11 with pembrolizumab (anti PD-1), one with durvalumab (anti-PD-L1), and one with atezolizumab (anti-PD-L1). One patient with MMP was treated with pembrolizumab.

Patients with a malignancy who started treatment with anti PD-1/ PD-L1 before MMP/BP onset may have a **suggestive** or **compatible** challenge. Arbitrarily, ≤32 weeks to BP onset was considered **suggestive** of anti PD-1/PD-L1 BP induction and ≥44 weeks was considered **compatible**. Thus, the challenge was **suggestive** in 20 patients (12 of the 14 patients with BP treated with nivolumab, seven of the 11 patients with BP treated with pembrolizumab, and one patient with MMP treated with pembrolizumab). It was **compatible** for the eight other patients.

The **dechallenge** was the discontinuation of the treatment. Twenty-one patients with BP discontinued anti PD-1/PD-L1 as follows: 11, nivolumab; eight, pembrolizumab; one, durvalumab; and one, atezolizumab. The reasons of this **dechallenge** are detailed in Table 2. Five patients did not discontinue anti PD-1/PD-L1 therapy. The information was not reported for the last case. Outcome after **dechallenge** or **no dechallenge** may be **suggestive** (if BP is controlled with **dechallenge** or worsened **without** **dechallenge),** conversely **nonsuggestive** (if BP worsened after **dechallenge** or controlled unless **without dechallenge**), and **inconclusive** (without details on BP evolution or continued treatment). Six of the 14 patients with BP treated with nivolumab had a **suggestive** outcome, seven had a **nonsuggestive** outcome, and one had an **inconclusive outcome**. Four of the 11 patients treated with pembrolizumab had a **suggestive** outcome; four, a **nonsuggestive;** and three, an **inconclusive** one. The two patients who were receiving anti-PD-L1 had a **suggestive** outcome. The patient with MMP had a suggestive challenge and dechallenge.

The **rechallenge** was the reintroduction of the treatment. It may be **positive** (R+) or **negative** (R-) or **not done** (R0). Two patients with BP had a **rechallenge,** one (patient 13) after control of the BP under oral corticosteroid and omalizumab with no reported relapse at **rechallenge** (R−) and another (patient 7) after 21 days of anti PD-1 discontinuation, who relapsed at **rechallenge** (R+). Twenty-five patients with BP and the patient with MMP were R0.

The **chronological** scoring (combining status of **challenge**, **dechallenge,** and **rechallenge**) may be C1, **doubtful**; C2, **plausible**; and C3, **likely**. Thirteen patients were C1; six, C2; and eight, C3.

The **symptomatological** scoring may be S1, **doubtful**; S2, **plausible**; and S3, **likely**. The seven patients with atypical clinical presentations were classified as S2. Twenty patients were S1.

Lastly, the **intrinsic accountability** scoring (combining **chronological** [C] and **symptomatological** [S] scores) may be I1 (C1S1), I2 (C1S2 or C2S1), I3 (C2S2), I4 (C1S3 or C3S1), I5 (C2S3 or C3S2), or I6 (C3S3). It was I5 for three, I4 for five, I3 for two, I2 for six, and I1 for 11 of the 27 patients with BP and I4 for the patient with MMP.

No marked differences were observed between the patients who received nivolumab and those who received pembrolizumab.
